# Interfacing Formate Dehydrogenase with Metal Oxides for the Reversible Electrocatalysis and Solar‐Driven Reduction of Carbon Dioxide

**DOI:** 10.1002/anie.201814419

**Published:** 2019-02-22

**Authors:** Melanie Miller, William E. Robinson, Ana Rita Oliveira, Nina Heidary, Nikolay Kornienko, Julien Warnan, Inês A. C. Pereira, Erwin Reisner

**Affiliations:** ^1^ Department of Chemistry University of Cambridge Cambridge CB2 1EW UK; ^2^ Instituto de Tecnologia Química e Biológica António Xavier Universidade Nova de Lisboa Av. da República 2780-157 Oeiras Portugal

**Keywords:** artificial photosynthesis, carbon dioxide fixation, formate dehydrogenase, interfaces, photocatalysis

## Abstract

The integration of enzymes with synthetic materials allows efficient electrocatalysis and production of solar fuels. Here, we couple formate dehydrogenase (**FDH**) from Desulfovibrio vulgaris Hildenborough (DvH) to metal oxides for catalytic CO_2_ reduction and report an in‐depth study of the resulting enzyme–material interface. Protein film voltammetry (PFV) demonstrates the stable binding of **FDH** on metal‐oxide electrodes and reveals the reversible and selective reduction of CO_2_ to formate. Quartz crystal microbalance (QCM) and attenuated total reflection infrared (ATR‐IR) spectroscopy confirm a high binding affinity for **FDH** to the TiO_2_ surface. Adsorption of **FDH** on dye‐sensitized TiO_2_ allows for visible‐light‐driven CO_2_ reduction to formate in the absence of a soluble redox mediator with a turnover frequency (TOF) of 11±1 s^−1^. The strong coupling of the enzyme to the semiconductor gives rise to a new benchmark in the selective photoreduction of aqueous CO_2_ to formate.

Electrocatalytic‐ and solar‐driven fuel synthesis from the greenhouse gas CO_2_ is a desirable approach to simultaneously produce sustainable energy carriers and combat increasing atmospheric CO_2_ levels.^1^ Formate is a stable intermediate in the reduction of CO_2_ and can be used as liquid energy carrier in fuel cells, as a hydrogen storage material, or feedstock for the synthesis of fine chemicals.^2^ Metals and synthetic molecular systems have been widely studied as electrocatalysts for CO_2_ reduction to formate, but largely lack the required efficiency, selectivity or affordability to enable carbon capture and utilization technologies.^3^, ^4^

There is avid research into both biological and artificial CO_2_ fixation. Semi‐artificial photosynthesis provides a common stage for these contrasting approaches as components from synthetic and biological origin can be combined in hybrid model systems.^5^ To date, enzyme‐based visible‐light‐driven CO_2_ reduction to formate relies on diffusional mediators, such as methyl viologen (MV^2+^) and nicotinamide adenine dinucleotide (NAD^+^).^6^, ^7^ Mediated processes are inefficient as they consume energy, are kinetically slow, and cause short‐circuit reactions. MV^2+^ is toxic to microorganisms,^8^ and NAD^+^ is prohibitively expensive for fuel production.^6^

In this work, we selected wild‐type formate dehydrogenase (**FDH**) from *Desulfovibrio vulgaris* Hildenborough (*Dv*H) as it has previously displayed robustness and high activity for the oxidation of formate in solution assays,^10^, ^11^ and the electrochemical reduction of CO_2_.^12^ Initially, protein film voltammetry (PFV) was employed to study the interfacial electron transfer between **FDH** and porous indium‐doped tin oxide (ITO) and TiO_2_ electrodes in the absence of a mediator. Immobilization and loading of **FDH** on TiO_2_ were then investigated using a quartz crystal microbalance (QCM) and attenuated total reflection infrared (ATR‐IR) spectroscopy. **FDH** was finally coupled directly to dye‐sensitized TiO_2_ nanoparticles for the selective photocatalytic reduction of CO_2_ to formate in a diffusional mediator‐free colloidal system (Figure 1).

**Figure 1:**
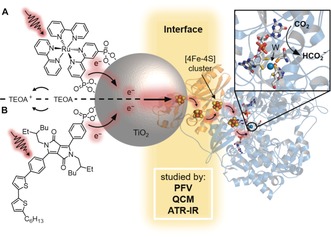
Schematic CO_2_ conversion with a dye–semiconductor–**FDH** photocatalyst system. Photoexcited electrons from the dye, **RuP** in (A) or **DPP** in (B), are transferred via the conduction band (CB) of TiO_2_ across the enzyme–material interface through the intraprotein [4Fe–4S] relays to the W‐active site of **FDH** for the reduction of CO_2_ to formate. The oxidized dye is regenerated by triethanolamine (TEOA). A protein structure homologous to *Dv*H **FDH** is shown.^9^

The electrocatalytic activity of **FDH** on metal‐oxide electrodes was studied by PFV on mesoporous ITO (*meso*ITO) and TiO_2_ (*meso*TiO_2_) electrodes with a film thickness of approximately 2.5 μm (Supporting Information, Figure S1).^13^ **FDH** (21.5 μm) was activated by incubation with the reducing agent dl‐dithiothreitol (DTT, 50 mm)^9^ and the resulting solution (2 μL) was drop‐cast on the electrode surface. The **FDH**‐modified electrode was placed in an electrolyte solution containing CO_2_/NaHCO_3_ and KCl at pH 6.5 under a CO_2_ atmosphere.

Figure 2 A shows the electrochemically reversible interconversion of CO_2_ and formate by **FDH** immobilized on a conductive *meso*ITO electrode (*meso*ITO|**FDH**). The onset potential for both CO_2_ reduction and formate oxidation was observed close to the thermodynamic potential (*E* ^0′^=−0.36 V vs. standard hydrogen electrode (SHE), pH 6.5),^14^ demonstrating that interfacial electron transfer by the [4Fe–4S] relays and catalysis at the W‐active site are highly efficient.^15^ Similar electrochemically reversible characteristics have previously only been reported for **FDH**s from *Escherichia coli* and *Syntrophobacter fumaroxidans* on graphite electrodes.^14^, ^16^, ^17^

**Figure 2:**
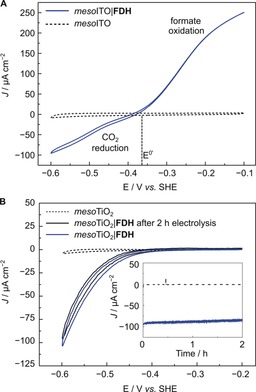
PFV (*ν*=5 mV s^−1^) showing A) reversible reduction of CO_2_ to formate by *meso*ITO|**FDH** (blue trace) and B) CO_2_ reduction by *meso*TiO_2_|**FDH** before (blue) and after 2 h CPE (black). Inset: CPE at −0.6 V vs. SHE. Conditions for A and B: 43 pmol **FDH** (amount drop‐cast), 100 mm CO_2_/NaHCO_3_, 50 mm KCl, 20 mm formate (only present in A), 1 atm CO_2_, pH 6.5, 25 °C, Pt counter electrode. Dashed traces show control experiments of **FDH**‐free electrodes.

When **FDH** was immobilized on a semiconducting *meso*TiO_2_ electrode (*meso*TiO_2_|**FDH**), a similar onset potential for CO_2_ reduction (−0.4 V vs. SHE) was observed and the current density reached −100 μA cm^−2^ at −0.6 V vs. SHE (Figure 2 B). Formate oxidation could not be observed for *meso*TiO_2_|**FDH** electrodes as TiO_2_ behaves as an insulator at the required potentials. Controlled‐potential electrolysis (CPE) at −0.6 V vs. SHE for 2 h produced formate with a Faradaic efficiency of (92±5)% (Figure 2 B, inset). Comparison of PFV scans before and after CPE showed that approximately 90 % of the initial **FDH** activity remains after 2 h, demonstrating the excellent stability of the immobilized enzyme.

The interaction of **FDH** and TiO_2_ was quantitatively investigated with a previously described QCM cell.^18^, ^19^ Upon flowing an **FDH**‐containing solution over a *planar*TiO_2_‐covered quartz chip (12 nm in 100 mm TEOA), the surface of TiO_2_ reached saturation after 1 h, resulting in approximately 3.5 pmol cm^−2^ of adsorbed **FDH** (*planar*TiO_2_|**FDH**, Figure 3 A). The strength of the enzyme–TiO_2_ interaction was probed by exposing the *planar*TiO_2_|**FDH** electrode to buffer solutions with different ionic strengths. Rinsing the QCM cell with an enzyme‐free solution for 1 h desorbed only 6 % of the preloaded **FDH**. Upon increasing the KCl concentration to 0.5–3.0 m KCl, 70–60 % of **FDH** remained adsorbed on the TiO_2_ surface. The finding that 60 % **FDH** remained adsorbed on TiO_2_ after multiple rinsing steps with high KCl concentrations suggests a contribution from chemisorption to the attachment of the enzyme.^20^, ^21^ Amino‐acid residues exposed on the **FDH** surface are likely involved in binding. For example, aspartic and glutamic acid have previously been suggested to form a strong interaction with TiO_2_.^22^, ^23^

**Figure 3:**
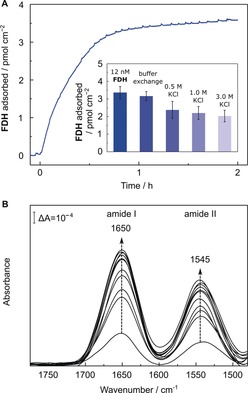
A) QCM analysis of the adsorption process of **FDH** on a *planar*TiO_2_‐coated quartz chip. Conditions: 12 nm **FDH**, 100 mm TEOA, open circuit potential of −0.1 to 0.0 V vs. SHE, pH 6.5, 25 °C, N_2_ atmosphere, circulation (0.141 mL min^−1^). Inset: Desorption of **FDH** by replacing the solution with fresh solution (100 mm TEOA) and subsequent increase of the ionic strength (each condition was held for 1 h). Error bars correspond to standard deviation (*N*=3). B) ATR‐IR absorbance spectra of the amide‐band region of **FDH** during the adsorption process over time onto a *planar*TiO_2_‐coated Si prism (100 nm thickness). Arrows indicate successively recorded spectra of every 1.5 min up to 7.5 min and subsequently every 30 min. Conditions: 1.0 μm **FDH**, 100 mm TEOA, total volume: 150 μL, open circuit potential, pH 6.5, 25 °C.

The adsorption of **FDH** was also probed by surface‐selective ATR‐IR spectroscopy using a Si prism coated with a *planar* or a *meso*TiO_2_ layer (100 or 400 nm thickness, respectively). After the addition of **FDH** to the buffer solution covering the *planar*TiO_2_ (Figure 3 B) or *meso*TiO_2_ (Supporting Information, Figure S2) coated prism, the two characteristic amide I and amide II bands of the protein backbone structure were detected at 1650 cm^−1^ and 1545 cm^−1^, respectively.^24^ The protein adsorption was monitored in situ over 2 h of incubation time and no (in the case of *planar*TiO_2_) or slight (in the case of *meso*TiO_2_) changes to the band features in the amide‐band region were observed, suggesting a mainly retained backbone structure of **FDH** on the surface of TiO_2_. During the adsorption process, amide I and amide II band intensities showed an increase over time (Figure 3 B). The majority of **FDH** remained adsorbed on the surface of *planar*TiO_2_ (Supporting Information, Figure S3) upon increasing the ionic strength of the buffer, which agrees with the QCM experiments (Figure 3 A, inset) and supports a stronger than purely electrostatic interaction between **FDH** and TiO_2_.

After establishing the strong interface between **FDH** and TiO_2_, visible‐light‐driven CO_2_ reduction to formate was investigated with **FDH** immobilized on dye‐sensitized TiO_2_ nanoparticles (dye|TiO_2_|**FDH**, Figures 1 and 4). The colloidal system was self‐assembled by adding **FDH** (pre‐activated with DTT) to a suspension of TiO_2_ nanoparticles containing TEOA and a phosphonate group‐bearing dye, either a ruthenium tris‐2,2′‐bipyridine complex (**RuP**) or a diketopyrrolopyrrole (**DPP**) at pH 6.5 and 25 °C under N_2_ atmosphere (to protect the enzyme from aerobic damage). Both dyes are known to adsorb onto TiO_2_ via their phosphonate‐anchoring groups and **DPP** provides a precious‐metal‐free alternative to **RuP**.^25^ CO_2_ was introduced to the solution via the addition of NaHCO_3_. Upon UV‐filtered irradiation, the photoexcited dye injects electrons into the conduction band (CB) of TiO_2_ (*E*_CB_(TiO_2_)=−0.67 V vs. SHE at pH 6.5),^25^ whereupon the electrons are conveyed to the catalytic W‐center of **FDH** to drive CO_2_ reduction. The oxidized dye is regenerated by the sacrificial electron donor (Figure 1).^26^

The dye|TiO_2_|**FDH** systems showed stable formate production for approximately 6 h (Figure 4). The formation of gaseous or dissolved side‐products was not detected by gas chromatography, ion chromatography, and ^1^H NMR spectroscopy. The activity of **RuP**|TiO_2_|**FDH** was not limited by the amount of dye or the light intensity (Supporting Information, Figures S4 and S5). A solution assay monitoring the activity of **FDH** by UV/Vis spectroscopy (via formate oxidation in presence of 2 mm MV^2+^) showed that approximately 36±7 % **FDH** remained active after 24 h of photocatalysis (Supporting Information, Figure S6), suggesting that inactivation of **FDH** is likely the main reason for activity loss. The addition of MV^2+^ as a soluble redox mediator to **RuP**|TiO_2_|**FDH** showed that not all **FDH** present in the system is accessible to direct electron transfer across the enzyme–material interface (Supporting Information, Figure S7). Control experiments demonstrated that all components are essential for formate production (Supporting Information, Figures S8 and S9) and support oxidative quenching and “through‐particle” electron transfer as depicted in Figure 1 (Supporting Information, Figures S10 and S11).^26^ Isotopic‐labeling studies confirmed that formate was produced from CO_2_ (Supporting Information, Figure S12).

**Figure 4:**
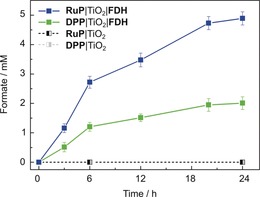
Photocatalytic CO_2_ reduction to formate with **FDH** in a colloidal dye‐sensitized TiO_2_ system. Conditions: 12 nm **FDH**, 10 mm DTT, 0.83 mg mL^−1^ TiO_2_, 16.7 μm dye (**RuP** or **DPP**), 100 mm TEOA, 100 mm NaHCO_3_, pH 6.5, 25 °C, total volume: 1.0 mL, assembled in an anaerobic glove box, UV‐filtered simulated solar‐light irradiation: 100 mW cm^−2^, AM 1.5G, *λ*>420 nm. Error bars correspond to standard deviation (*N*=3). Dashed traces show control experiments in the absence of **FDH**.

For photocatalytic experiments, an enzyme loading of approximately 0.03 pmol cm^−2^ was calculated assuming that all **FDH** is adsorbed on TiO_2_ with a surface area of 50 m^2^ g^−1^. Saturation of the TiO_2_ surface with **FDH** in the QCM experiment was only observed when two orders of magnitude higher amounts of **FDH** were adsorbed (Figure 3 A). As QCM and ATR‐IR spectroscopy indicate stronger than purely electrostatic interactions, close‐to‐quantitative adsorption of **FDH** on the TiO_2_ nanoparticle in the colloidal system is likely. A turnover frequency (TOF) of 11±1.0 and 5±0.6 s^−1^ (based on CO_2_ conversion after 6 h) and approximately 4.9±0.2 and 2.0±0.2 μmol formate (after 24 h) were observed from CO_2_ using **RuP** and **DPP**‐sensitized TiO_2_, respectively (Figure 4). The results of all photocatalysis experiments are presented in Tables S1 and S2 in the Supporting Information.

Table 1 shows a comparison of state‐of‐the‐art catalysts (enzymatic and synthetic) in combination with dye‐sensitized TiO_2_ nanoparticles without diffusional mediators for CO_2_ reduction and H_2_ evolution. Previous studies showed that enzymes outperform the synthetic systems in terms of TOF.^30^ Among the compared systems, the presented **RuP**|TiO_2_|**FDH** system exhibits the highest TOF for CO_2_ reduction. The **DPP**|TiO_2_|**FDH** system shows that comparable activities can also be achieved in an entirely precious‐metal‐free system. In semi‐artificial systems, rapid electron transfer from TiO_2_ to the enzyme was previously found to be essential for efficient catalysis,^22^, ^31^ suggesting that the strong interfacial interaction plays an important role for the high activity and stability of dye|TiO_2_|**FDH**. Previously reported photocatalyst systems employing NAD^+^‐dependent **FDH**s for CO_2_ reduction to formate rely on soluble redox mediators and only produced TOFs in the range of 10–20 h^−1^.^32^

**Table 1:** Comparison of TOFs for dye‐sensitized TiO_2_ systems with enzymatic and synthetic catalysts for CO_2_ reduction and H_2_ evolution.

reaction	dye	catalyst	TOF [h^−1^]	ref.
CO_2_ → HCO_2_^−^	**RuP**	*DvH* **FDH** ^[a]^	4.0×10^4^	this work
	**DPP**	*DvH* **FDH** ^[a]^	1.8×10^4^	this work
				
CO_2_ → CO	**RuP**	*Ch* CODH I^[b]^	5.4×10^2^	27
	dye^[c]^	**Re** ^[d]^	8.6	28
				
H^+^ → H_2_	**RuP**	*Db* [NiFeSe]‐H_2_ase^[e]^	1.8×10^5^	22
	**DPP**	*Db* [NiFeSe]‐H_2_ase^[e]^	8.7×10^3^	25
	CN_*x*_^[f]^	*Db* [NiFeSe]‐H_2_ase^[e]^	2.8×10^4^	23
	**RuP**	**Ni** ^[g]^	3.2×10^2^	29

[a] W‐**FDH** from *Dv*H. [b] Carbon monoxide dehydrogenase (CODH) I from *Carboxydothermus hydrogenoformans* (*Ch*). [c] (*E*)‐2‐cyano‐3‐(5′‐(5′′‐(*p*‐(diphenylamino)phenyl)thiophen‐2′′‐yl)thiophen‐2′‐yl)‐acrylic acid. [d] Synthetic rhenium catalyst (**Re**) in *N*,*N*‐dimethyl formamide (DMF) and water. [e] [NiFeSe]‐hydrogenase from *Desulfomicrobium baculatum* (*Db*). [f] Polyheptazine carbon nitride polymer melon (CN_*x*_). [g] Synthetic nickel(II) bis(diphosphine) catalyst (**Ni**).

In summary, **FDH** immobilized on metal‐oxide electrodes is established as a reversible electrocatalyst for the selective conversion of CO_2_ to formate. The porous metal‐oxide scaffolds allow for high **FDH** loading and consequently high current densities, which makes the protein‐modified electrodes not only a relevant model system for CO_2_ utilization, but also for formate oxidation in formate fuel cells. An excellent interface between TiO_2_ and **FDH** is confirmed by QCM analysis and ATR‐IR spectroscopy. The direct (diffusional mediator‐free) electron transfer across the enzyme–metal‐oxide interface is exploited for visible‐light‐driven CO_2_ reduction to formate. These results underline the importance of characterizing the interactions at the enzyme–material interface and future improvements in performance may arise from more controlled immobilization and more efficient electron transfer with the directly wired **FDH**.

## Conflict of interest

The authors declare no conflict of interest.

## Supporting information

As a service to our authors and readers, this journal provides supporting information supplied by the authors. Such materials are peer reviewed and may be re‐organized for online delivery, but are not copy‐edited or typeset. Technical support issues arising from supporting information (other than missing files) should be addressed to the authors.

Supplementary

## References

[1] D. G. Nocera , Acc. Chem. Res. 2017, 50, 616–619. 28945407 10.1021/acs.accounts.6b00615

[2] A. Boddien , D. Mellmann , F. Gärtner , R. Jackstell , H. Junge , P. J. Dyson , G. Laurenczy , R. Ludwig , M. Beller , Science 2011, 333, 1733–1736. 21940890 10.1126/science.1206613

[3] S. Gao , Y. Lin , X. Jiao , Y. Sun , Q. Luo , W. Zhang , D. Li , J. Yang , Y. Xie , Nature 2016, 529, 68–71. 26738592 10.1038/nature16455

[4] K. E. Dalle , J. Warnan , J. J. Leung , B. Reuillard , I. S. Karmel , E. Reisner , Chem. Rev. 2019, in print (DOI: 10.1021/acs.chemrev.8b00392). 10.1021/acs.chemrev.8b00392 PMC6396143 30767519

[5] N. Kornienko , J. Z. Zhang , K. K. Sakimoto , P. Yang , E. Reisner , Nat. Nanotechnol. 2018, 13, 890–899. 30291349 10.1038/s41565-018-0251-7

[6] J. Kim , S. H. Lee , F. Tieves , D. S. Choi , F. Hollmann , C. E. Paul , C. B. Park , Angew. Chem. Int. Ed. 2018, 57, 13825–13828; 10.1002/anie.201804409 30062834

[7] B. A. Parkinson , P. F. Weaver , Nature 1984, 309, 148–149.

[8] S. F. Rowe , G. Le Gall , E. V. Ainsworth , J. A. Davies , C. W. J. Lockwood , L. Shi , A. Elliston , I. N. Roberts , K. W. Waldron , D. J. Richardson , et al., ACS Catal. 2017, 7, 7558–7566.

[9] H. Raaijmakers , S. Macieira , J. M. Dias , S. Teixeira , S. Bursakov , R. Huber , J. J. G. Moura , I. Moura , M. J. Romão , Structure 2002, 10, 1261–1272. 12220497 10.1016/s0969-2126(02)00826-2

[10] S. M. da Silva , C. Pimentel , F. M. A. Valente , C. Rodrigues-Pousada , I. A. C. Pereira , J. Bacteriol. 2011, 193, 2909–2916. 21498650 10.1128/JB.00042-11 PMC3133204

[11] S. M. da Silva , J. Voordouw , C. Leitão , M. Martins , G. Voordouw , I. A. C. Pereira , Microbiology 2013, 159, 1760–1769. 23728629 10.1099/mic.0.067868-0

[12] K. P. Sokol , W. E. Robinson , A. R. Oliveira , J. Warnan , M. M. Nowaczyk , A. Ruff , I. A. C. Pereira , E. Reisner , J. Am. Chem. Soc., 2018, 140, 16418–16422. 30452863 10.1021/jacs.8b10247 PMC6307851

[13] M. Kato , T. Cardona , A. W. Rutherford , E. Reisner , J. Am. Chem. Soc. 2012, 134, 8332–8335. 22548478 10.1021/ja301488d

[14] T. Reda , C. M. Plugge , N. J. Abram , J. Hirst , Proc. Natl. Acad. Sci. U. S. A. 2008, 105, 10654–10658. 18667702 10.1073/pnas.0801290105 PMC2491486

[15] F. A. Armstrong , J. Hirst , Proc. Natl. Acad. Sci. U. S. A. 2011, 108, 14049–14054. 21844379 10.1073/pnas.1103697108 PMC3161523

[16] A. Bassegoda , C. Madden , D. W. Wakerley , E. Reisner , J. Hirst , J. Am. Chem. Soc. 2014, 136, 15473–15476. 25325406 10.1021/ja508647u

[17] W. E. Robinson , A. Bassegoda , E. Reisner , J. Hirst , J. Am. Chem. Soc. 2017, 139, 9927–9936. 28635274 10.1021/jacs.7b03958 PMC5532686

[18] N. Kornienko , N. Heidary , G. Cibin , E. Reisner , Chem. Sci. 2018, 9, 5322–5333. 30009004 10.1039/c8sc01415a PMC6009440

[19] D. H. Nam , J. Z. Zhang , V. Andrei , N. Kornienko , N. Heidary , A. Wagner , K. Nakanishi , K. P. Sokol , B. Slater , I. Zebger , et al., Angew. Chem. Int. Ed. 2018, 57, 10595–10599; 10.1002/anie.201805027 PMC6100105 29888857

[20] S. Frasca , T. von Graberg , J.-J. Feng , A. Thomas , B. M. Smarsly , I. M. Weidinger , F. W. Scheller , P. Hildebrandt , U. Wollenberger , ChemCatChem 2010, 2, 839–845.

[21] H. K. Ly , M. A. Marti , D. F. Martin , D. Alvarez-Paggi , W. Meister , A. Kranich , I. M. Weidinger , P. Hildebrandt , D. H. Murgida , ChemPhysChem 2010, 11, 1225–1235. 20376873 10.1002/cphc.200900966

[22] E. Reisner , D. J. Powell , C. Cavazza , J. C. Fontecilla-Camps , F. A. Armstrong , J. Am. Chem. Soc. 2009, 131, 18457–18466. 19928857 10.1021/ja907923r

[23] C. A. Caputo , L. Wang , R. Beranek , E. Reisner , Chem. Sci. 2015, 6, 5690–5694. 28757952 10.1039/c5sc02017d PMC5512016

[24] A. Barth , Biochim. Biophys. Acta Bioenerg. 2007, 1767, 1073–1101. 10.1016/j.bbabio.2007.06.004 17692815

[25] J. Warnan , J. Willkomm , J. N. Ng , R. Godin , S. Prantl , J. R. Durrant , E. Reisner , Chem. Sci. 2017, 8, 3070–3079. 28451376 10.1039/c6sc05219c PMC5380916

[26] J. Willkomm , K. L. Orchard , A. Reynal , E. Pastor , J. R. Durrant , E. Reisner , Chem. Soc. Rev. 2016, 45, 9–23. 26584204 10.1039/c5cs00733j

[27] T. W. Woolerton , S. Sheard , E. Pierce , S. W. Ragsdale , F. A. Armstrong , Energy Environ. Sci. 2011, 4, 2393–2399.

[28] J.-S. Lee , D.-I. Won , W.-J. Jung , H.-J. Son , C. Pac , S. O. Kang , Angew. Chem. Int. Ed. 2017, 56, 976–980; 10.1002/anie.201608593 27966811

[29] M. A. Gross , A. Reynal , J. R. Durrant , E. Reisner , J. Am. Chem. Soc. 2014, 136, 356–366. 24320740 10.1021/ja410592d PMC3901378

[30] C. Wombwell , C. A. Caputo , E. Reisner , Acc. Chem. Res. 2015, 48, 2858–2865. 26488197 10.1021/acs.accounts.5b00326

[31] T. W. Woolerton , S. Sheard , E. Reisner , E. Pierce , S. W. Ragsdale , F. A. Armstrong , J. Am. Chem. Soc. 2010, 132, 2132–2133. 20121138 10.1021/ja910091z PMC2845288

[32] S. H. Lee , D. S. Choi , S. K. Kuk , C. B. Park , Angew. Chem. Int. Ed. 2018, 57, 7958–7985; 10.1002/anie.201710070 29194901

