# Supplementary material for: Interfacing Formate Dehydrogenase with Metal Oxides for the Reversible Electrocatalysis and Solar‐Driven Reduction of Carbon Dioxide
Source: Angew Chem Int Ed Engl. 2019 Feb 22;58(14):4601–5. doi: 10.1002/anie.201814419 (PMC6563039; doi:10.1002/anie.201814419)
Supplement: Supplementary file 1 — Supplementary [file ANIE-58-4601-s001.pdf]

Angewandte Chemie
Eine Zeitschrift der Gesellschaft Deutscher Chemiker

Supporting Information

Interfacing Formate Dehydrogenase with Metal Oxides for the Reversible Electrocatalysis and Solar-Driven Reduction of Carbon Dioxide

*Melanie Miller, William E. Robinson, Ana Rita Oliveira, Nina Heidary, Nikolay Kornienko, Julien Warnan, Inês A. C. Pereira, and Erwin Reisner**

anie_201814419_sm_miscellaneous_information.pdf

Experimental Section

Materials. The following chemicals and materials were obtained from commercial suppliers and used without further purification unless otherwise stated: acetic acid (Fisher Chemical), ammonium sulfate (Panreac), carbon dioxide gas (BOC), deuterium oxide (Sigma Aldrich, 99.9 atom % D), dipotassium hydrogenphosphate (Honeywell), *DL*-dithiothreithol (DTT, Sigma Aldrich), ethanol (VWR Chemicals), glycerol (Scharlau), indium-doped tin oxide (ITO, Sigma Aldrich, < 50 nm diameter), isopropanol (Honeywell), ITO-coated glass slides (Sigma Aldrich, 8-12 Ω sq^{-1}), methyl viologen dichloride hydrate (MV^{2+}, Sigma Aldrich, 98%), Nafion® (Sigma Aldrich), Parafilm® M (Sigma Aldrich), potassium chloride (Fisher Chemical), potassium dihydrogen phosphate (Panreac), rubber septa (Subaseal), sodium hydrogen carbonate (Fisher Scientific), sodium hydrogen carbonate-^{13}C (Sigma Aldrich, 98 atom % ^{13}C), sodium carbonate (Fisher Chemical), sodium formate (Sigma Aldrich, ≥ 99.0%), sodium formate-^{13}C (Sigma Aldrich, 99 atom % ^{13}C), sodium hydroxide (Fisher Chemical), sodium nitrate (Panreac), sulfuric acid (Fisher Chemical, > 95%), triethanolamine (TEOA, Sigma Aldrich), tris(hydroxymethyl)aminomethane (Tris, Sigma Aldrich), titanium dioxide (Evonik Industries, P25, 80:20 anatase : rutile, 21 nm diameter), 3-(trimethylsilyl)propionic-2,2,3,3-d_4 acid sodium salt (TSP, Sigma Aldrich, 98 atom % D), zirconium dioxide (SkySpring Nanomaterials, Inc., 99.99%, 20-30 nm diameter). Sodium formate was dried under high vacuum at 100°C for 3 h before use. The following compounds were synthesised as reported previously: diketopyrrolopyrrole (**DPP**)[1], ruthenium tris-2,2'-bipyridine complex (**RuP**).[2] Tungsten-containing formate dehydrogenase (**FDH**) from *Desulfovibrio vulgaris* Hildenborough (*Dv*H) was purified with some modifications to the previous reports.[3,4] The soluble fraction of *Dv*H cells grown in the presence of Na_2WO_4 x H_2O was loaded on a Q-sepharose high performance column (GE Healthcare), equilibrated with 20 mM Tris-HCl pH 7.6 with 10% (v/v) glycerol and 10 mM $NaNO_3$. Elution was performed with stepwise increments on NaCl concentration. The buffer of the fractions containing **FDH** activity, eluted around 150 mM NaCl, was exchanged to 20 mM KPi (K_2HPO_4/KH_2PO_4) buffer with 10% (v/v) glycerol, and 10 mM $NaNO_3$ (buffer K). Then 15% (w/v) $(NH_4)_2SO_4$ was added to the sample, which was further purified using a phenyl sepharose high performance column (GE Healthcare) equilibrated with buffer K with 15% (v/v) $(NH_4)_2SO_4$. Elution was performed by reducing the ionic strength in a stepwise decreasing gradient. Pure **FDH** was eluted between 10.5 and 12% (v/v) $(NH_4)_2SO_4$ and the buffer was exchanged to 20 mM Tris-HCl, 10% glycerol, 10 mM $NaNO_3$, pH 7.6. All purification steps were performed under aerobic conditions at 4ºC. 43 µM **FDH** stock solutions with an activity of 1100 s^{-1} for formate oxidation and 320 s^{-1} for CO_2 reduction were stored in a buffer solution (20 mM Tris-HCl, 10% glycerol, 10 mM $NaNO_3$, pH 7.6) at −40°C under N_2 atmosphere. Millipore water (18.2 MΩ cm at 25°C) was used throughout this work.

Preparation of *meso*ITO|FDH and *meso*TiO$_2$|FDH electrodes. Mesoporous metal oxide films (geometrical surface area A = 0.25 cm^{-2}) on conducting indium-doped tin oxide (ITO) coated glass (2 × 1 cm^2) were prepared as previously reported.[5–7] The glass slides were cleaned by sonication in isopropanol (2 × 15 min), ethanol (2 x 15 min), and rinsing with H_2O. After drying at 150°C overnight, parafilm masks with a hole (5 mm diameter) were attached to the glass slides before 5 µL of a suspension of ITO (20wt% ITO in an acetic acid solution in 5 M EtOH) or TiO_2 nanoparticles (100 mg TiO_2 and 50 mg poly(ethylene glycol) in 1.0 mL EtOH) was deposited on top by doctor blading. After drying in air, the slides were annealed at 400°C for 1 h (*meso*ITO) or at 450°C for 0.5 h (*meso*TiO_2) with a heating rate of 4°C min^{-1}. **FDH** was pre-incubated (5 min) with DTT (2 µl, 21.5 µM **FDH**, 50 mM DTT) before immobilization on *meso*ITO or *meso*TiO_2 electrodes under N_2 atmosphere.

Protein film voltammetry. A gas-tight two compartment cell with a Nafion membrane separating the compartments was equipped with a three-electrode setup, consisting of a Ag/AgCl (saturated KCl) reference electrode (−0.199 V *vs.* standard hydrogen electrode (SHE)), a Pt mesh counter electrode, and a *meso*ITO|**FDH** or *meso*TiO_2|**FDH** working electrode. An electrolyte solution containing CO_2/$NaHCO_3$ (100 mM) and KCl (50 mM) at pH 6.5 was prepared by dissolving $NaHCO_3$ and KCl in H_2O and purging with CO_2 for 15 min. The electrochemical cell was filled with electrolyte, sealed with rubber septa, constantly kept at 25°C, and purged again with CO_2 for 5 min before the start of the measurement. All electrochemical experiments were performed with an Ivium CompactStat potentiostat and voltammograms were recorded with a scan rate of 5 mV s^{-1} under continuous stirring. Chronoamperometry was performed at −0.6 V *vs.* SHE. All data processing was performed using OriginPro 9.1 software.

Assembly of the photocatalytic system. In a standard experiment, TiO_2 (0.83 mg mL^{-1}) was dispersed in TEOA (100 mM, pH 6.5) *via* sonication (10 min), **RuP** (16.7 µM) was added and the mixture stirred (10 min) in the dark before the addition of $NaHCO_3$ (100 mM). **FDH** (12 nM) was pre-incubated (5 min) with DTT (100 µL, 375 mM DTT, 450 nM **FDH**, 20 mM Tris) before addition. The photoreactors, containing a total volume of 1.0 mL, were sealed with a rubber septum before they were placed in the solar light simulator and irradiation (100 mW cm^{-2}, AM 1.5G, λ > 420nm, 25°C, N_2 atmosphere) was started. All experiments were performed under continuous stirring as triplicate runs. Unless otherwise stated, all photocatalytic experiments were assembled as described above. Usually, liquid samples with a volume of 20 µL were taken and analysed by ion chromatography (see below). All data processing was performed using OriginPro 9.1 software. Errors bars are ± sample standard deviation (s)

estimated from three experiments. Control experiments shown in Figure S8 were normalised relative to the activity of the complete system due to the use of a different enzyme batch with a different activity.

Quartz crystal microbalance analysis. Quartz crystal microbalance (QCM) experiments were conducted with a Biolin Q-Sense Explorer module and a custom-designed QCM electrochemical cell. Typically, a titanium-coated quartz chip, featuring a native oxide titania surface was cleaned through sonication in Hellmanex surfactant (1% vol. in deionized water) and deionized water for 15 min each, followed by a 15 min UV-ozone treatment. Prior to measuring, 10 mL of enzyme-free TEOA buffer solution (100 mM), under a nitrogen environment, was flowed through at 0.141 mL min^{-1} for 1-2 h to generate a stable baseline. Following this, a small aliquot of enzyme was added in to reach a final concentration of 12 nM. Enzyme adsorption was quantified by monitoring changes in the resonance frequency of the piezoelectric quartz chip. The frequency was related to the mass through the Sauerbrey equation:

$$\Delta f = -\frac{2f_0^2}{A\sqrt{p_q \mu_q}}\Delta m$$

where f_0 is the resonance frequency of the quartz oscillator, A is the piezoelectrically active crystal area, Δm is the change in mass, p_q is the density of quartz, and μ_q is the shear modulus of quartz. To convert the mass adsorbed to quantity of enzyme, an assumption was made that 25% of the adsorbed mass consisted of water molecules bound to the enzyme, which was 143 kDa in weight.

Attenuated total reflection infrared spectroscopy. Attenuated total reflection infrared spectroscopy (ATR-IR) measurements were performed in a single-reflection PIKE ATR-IR setup and a customized ATR-cell using a Si prism with an angle of incidence of 60°. In order to probe **FDH** immobilization and surface interaction, planar and mesoporous TiO_2 films were spin-coated onto the flat Si surface. Thin films were fabricated by a sol-gel process for planar TiO_2 films[8] and for mesoTiO_2 films as mentioned above. Film thicknesses of 100 nm and 400 nm were obtained for planar and mesoporous TiO_2 respectively. ATR-IR spectra were recorded from 4000 to 1000 cm^{-1} with a spectral resolution of 4 cm^{-1} on a Bruker Vertex 70 spectrometer equipped with a photovoltaic MCT detector. Two hundred scans were co-added for one spectrum, requiring an accumulation time of 1.5 min. ATR-IR spectra were evaluated using OPUS 5.5 software. Immobilization of the **FDH** was accomplished by incubating a 43 µM of **FDH** solution (4 µL) added to a 146 µL of TEOA buffer solution (100 mM) at pH 6.5 for 2h (planar TiO_2) and 30 min (mesoTiO_2) at room temperature.

Ion chromatography. Ion chromatography (IC) was carried out on a Metrohm 882 Compact IC Plus ion chromatograph with a conductivity detector. The eluent buffer was an aqueous solution of Na_2CO_3 (3 mM), $NaHCO_3$ (1 mM), and acetone (50 mL L^{-1}). Each time, after renewing the eluent buffer, the system was calibrated with samples containing 6.25, 12.5, 25, 37.5, 50, 75, and 100 µM sodium formate. Samples were diluted 50 x with H_2O and filtered before injection in the IC.

Scanning electron microscopy. Scanning electron microscopy (SEM) was carried out on a TESCAN MIRA3 FEG-SEM. Cross sections of the electrodes were prepared and sputtered with a 10 µm layer of Pt prior to the measurement.

Nuclear magnetic resonance spectroscopy. ^{1}H spectra were recorded on a Bruker Avance III 400 MHz spectrometer (equipped with a BBO probe) in D_2O using water suppression by presaturation at room temperature. Chemical shifts are referenced relative to the protium/^{1}H signal of TSP: δ = 0.00 ppm.

Solution assay for activity determination via UV-vis spectroscopy. The formate oxidation activity of **FDH** in the colloidal system before and after 24 h of irradiation was determined via a UV-vis spectroscopic assay. Absorption spectra were measured at λ = 578 nm by a Varian Cary 50 Bio spectrometer using quartz cuvettes (Hellma, 1 cm pathlength). A typical photocatalysis sample (see above) was diluted 50 x with H_2O and sodium formate was added to reach a final concentration of 10 mM. 1.0 mL of the solution was transferred in a cuvette under N_2 atmosphere and placed in the spectrophotometer. After 1 min, the formate oxidation was started by the injection of methyl viologen (MV^{2+}, 2 mM) and the change in absorbance at λ = 578 nm was monitored over time (Figure S6).

Table S1. Amount of formate produced (24 h), turnover number (24 h), and turnover frequency (6 h) including sample standard deviations (± s) for photocatalytic CO_2 reduction to formate with **FDH** in a colloidal dye-sensitized TiO_2 system. Conditions: 12 nM **FDH**, 10 mM DTT, 0.83 mg mL^{-1} TiO_2, 16.7 µM **RuP**, 100 mM TEOA, 100 mM $NaHCO_3$, pH 6.5, 25°C, N_2 atmosphere, total volume: 1.0 mL, simulated solar light irradiation: 100 mW cm^{-2}, AM 1.5G, λ > 420 nm.

#	System	Formate ± s / µmol (24 h)	TON **FDH** ± s / mol formate (mol **FDH**)$^{-1}$ (24 h)	TOF ± s / s^{-1} (6 h)
1	**RuP**|TiO_2|**FDH**	4.9 ± 0.2	409 000 ± 18 700	11 ± 0.8
2	**DPP**|TiO_2|**FDH**	2.0 ± 0.2	169 000 ± 18 100	5 ± 0.6

Table S2. Control experiments: Amount of formate produced (24 h), and turnover number (24 h) including sample standard deviations (± s) for photocatalytic CO_2 reduction to formate with **FDH** in a colloidal dye-sensitized TiO_2 system. Conditions: 12 nM **FDH**, 10 mM DTT, 0.83 mg mL^{-1} TiO_2, 16.7 µM **RuP**, 100 mM TEOA, 100 mM $NaHCO_3$, pH 6.5, 25°C, N_2 atmosphere, total volume: 1.0 mL, simulated solar light irradiation: 100 mW cm^{-2}, AM 1.5G, λ > 420 nm. The difference to the standard experiment is indicated in the column labelled change.

#	System	Change	Formate ± s / µmol (24 h)	TON **FDH** ± s / mol formate (mol **FDH**)$^{-1}$ (24 h)
3	**RuP**|TiO_2	no **FDH**	0	0
4	**DPP**|TiO_2	no **FDH**	0	0
5	**RuP**|TiO_2|**FDH**	no TEOA[a]	5.8 ± 0.3	485 000 ± 21 500
6	TiO_2|**FDH**	no **dye**	0.4 ± 0.1	34 900 ± 5 220
7	**RuP**|TiO_2|**FDH**	no DTT	0.7 ± 0.1	54 300 ± 7 480
8	**RuP**|TiO_2|**FDH**	no $NaHCO_3$	0.3 ± 0.1	28 700 ± 9 430
9	**RuP**|TiO_2|**FDH**	no TiO_2	0	0
10	**RuP**|TiO_2|**FDH**	no light	0	0
11	**RuP**|ZrO_2|**FDH**	TiO_2 replaced by ZrO_2	0	0
12	**DPP**|ZrO_2|**FDH**	TiO_2 replaced by ZrO_2	0	0
13	**RuP**|TiO_2|**FDH**	centrifugation and washing in TEOA buffer[b]	1.4 ± 0.3	119 000 ± 26 900
14	**RuP**|TiO_2 |**FDH**	centrifugation and washing in carbonate buffer[b]	0	0
15	TiO_2 |**FDH**	full solar light irradiation	4.3 ± 0.3	357 000 ± 22 800
16	**RuP**|TiO_2 |**FDH**	addition of **RuP** after 24 h	4.3 ± 0.4	359 000 ± 35 500
17	**RuP**|TiO_2 |**FDH**	50 and 80% neutral density filters	4.2 ± 0.3	349 000 ± 26 100

[a] DTT acts as sacrificial electron donor in the absence of TEOA (Figures S8 and S9).
[b] See Figure S9 for details.

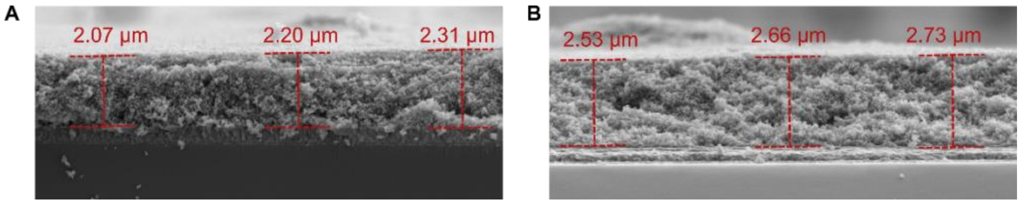

Figure S1. Cross-sectional SEM images of (A) *meso*ITO and (B) *meso*TiO_2 electrodes on ITO-coated class slides. Synthetic procedures can be found in the Experimental Section.

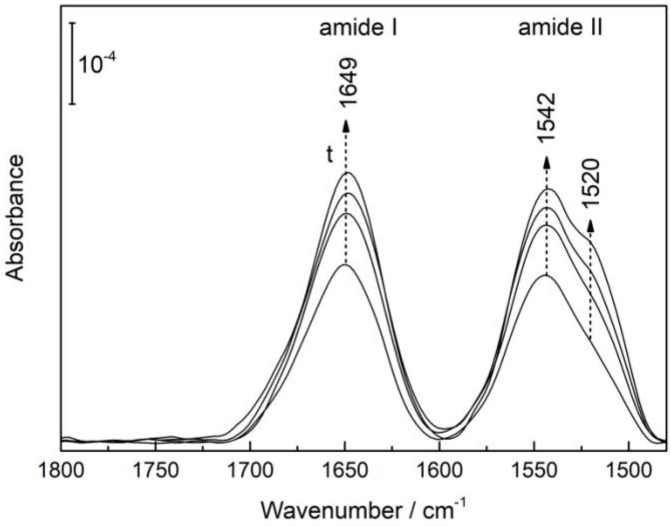

Figure S2. ATR-IR absorbance spectra of the amide band region of **FDH** during the adsorption process over time onto *meso*TiO_2 (400 nm thickness). Conditions: 1.0 µM **FDH,** 100 mM TEOA, total volume: 150 µL, open circuit potential, pH 6.5, 25°C. Arrows indicate successive recorded spectra of every 7.5 min with the first spectrum shown after 7.5 min of immobilization time. The amide I and amide II band show the similar main amide band features as observed on *planar*TiO_2 (Figure 3B). The shift of amide I and amide II band positions of 1 cm^{-1} and 3 cm^{-1} to lower wavenumbers lie within the resolution of the spectra (4 cm^{-1}). A shoulder band at 1520 cm^{-1}, which is more pronounced during **FDH** immobilization on the *meso*TiO_2 electrode than on *planar*TiO_2, was observed. This might be an indication for a small change in the **FDH** secondary structure induced by its interaction within the 3D framework.

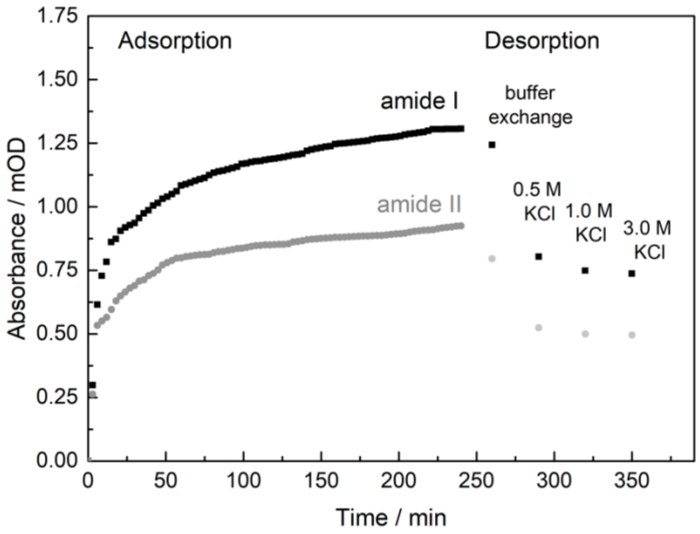

Figure S3. ATR-IR band intensities of amide I and amide II (1650 cm^{-1} and 1545 cm^{-1}) as a function of immobilization time for the adsorption process of **FDH** onto *planar*TiO_2 (100 nm thickness). The corresponding absorbance spectra of the amide band region are shown in Figure 3B. Conditions: 1.0 µM **FDH,** 100 mM TEOA, total volume: 150 µL, open circuit potential, pH 6.5, 25°C. Desorption of **FDH** from the surface was induced by replacing the **FDH**-containing solution with buffer solutions of increasing ionic strength (all containing 100 mM TEOA and KCl as indicated in the figure). Each condition was held for 30 min and ATR-IR spectra were recorded in 100 mM TEOA. All ATR-IR spectra were referenced to 100 mM TEOA buffer on *planar*TiO_2.

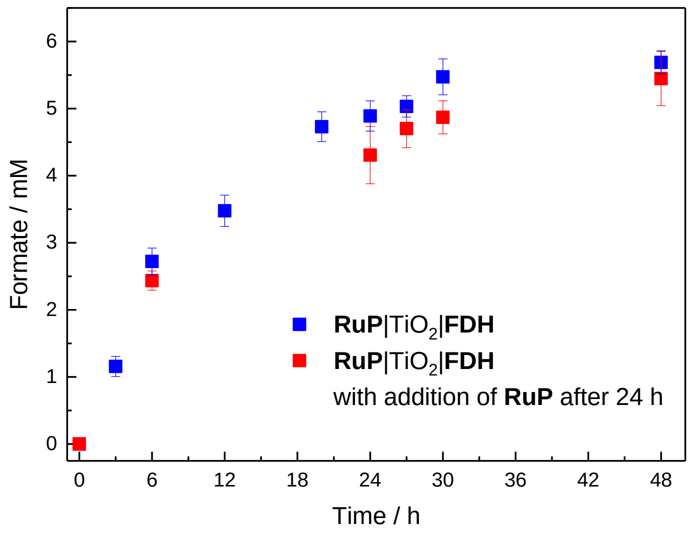

Figure S4. The effect of adding additional **RuP** (16.7 µM, red) after 24 h of solar light irradiation in a colloidal dye-sensitized TiO_2 system for photocatalytic CO_2 reduction to formate with **FDH**. Conditions: 12 nM **FDH**, 10 mM DTT, 0.83 mg mL^{-1} TiO_2, 16.7 µM **RuP**, 100 mM TEOA, 100 mM $NaHCO_3$, pH 6.5, 25°C, N_2 atmosphere, total volume: 1.0 mL, simulated solar light irradiation: 100 mW cm^{-2}, AM 1.5G, $\lambda > 420$ nm. Error bars correspond to sample standard deviation ($N = 3$). Data for **RuP**|TiO_2|**FDH** (blue) up to 24 h was reproduced from Figure 4.

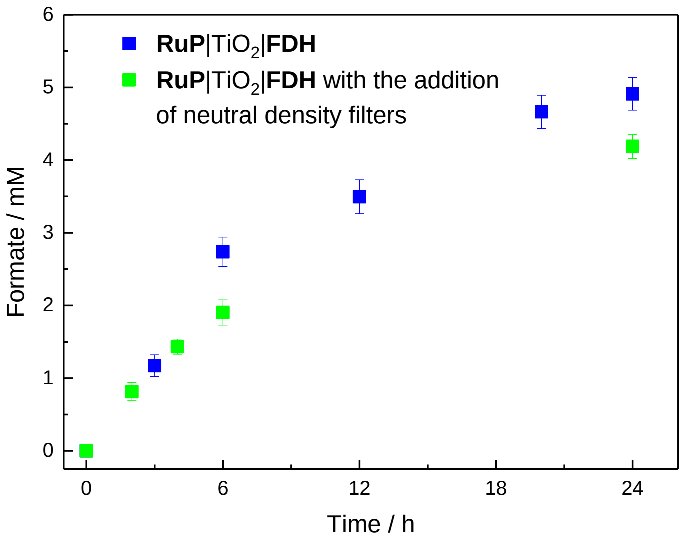

Figure S5. The effect of light intensity on formate production over time in a colloidal dye-sensitized TiO_2 system for photocatalytic CO_2 reduction to formate with **FDH**. Conditions: 12 nM **FDH**, 10 mM DTT, 0.83 mg mL^{-1} TiO_2, 16.7 µM **RuP**, 100 mM TEOA, 100 mM $NaHCO_3$, pH 6.5, 25°C, N_2 atmosphere, total volume: 1.0 mL, simulated solar light irradiation: 100 mW cm^{-2}, AM 1.5G, $\lambda > 420$ nm. Addition of neutral density filters: 50% after 2 h and 80% after 4 h (green). Error bars correspond to sample standard deviation ($N = 3$). Data for **RuP**|TiO_2|**FDH** (blue) up to 24 h was reproduced from Figure 4.

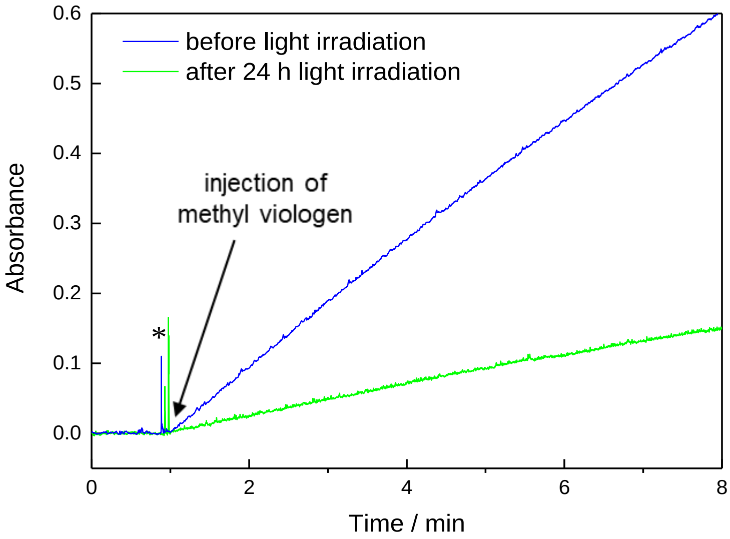

Figure S6. Formate oxidation with **FDH** before (blue) and after 24 h (green) of UV-filtered simulated solar light irradiation. 2 mM MV^{2+} was injected in a UV-vis cuvette containing the corresponding sample after 1 min and the MV^{2+} reduction was monitored by observing the increase in absorbance at $\lambda = 578$ nm (corresponding to MV^{+}). Conditions: 12 nM **FDH**, 10 mM DTT, 0.83 mg mL^{-1} TiO_2, 16.7 µM **RuP**, 100 mM TEOA, 100 mM $NaHCO_3$, pH 6.5, 25°C, N_2 atmosphere, total volume: 1.0 mL, UV-filtered simulated solar light irradiation: 100 mW cm^{-2}, AM 1.5G, $\lambda > 420$ nm. * The artefacts are a result of the disturbance of the measurement caused by the injection of MV^{2+}.

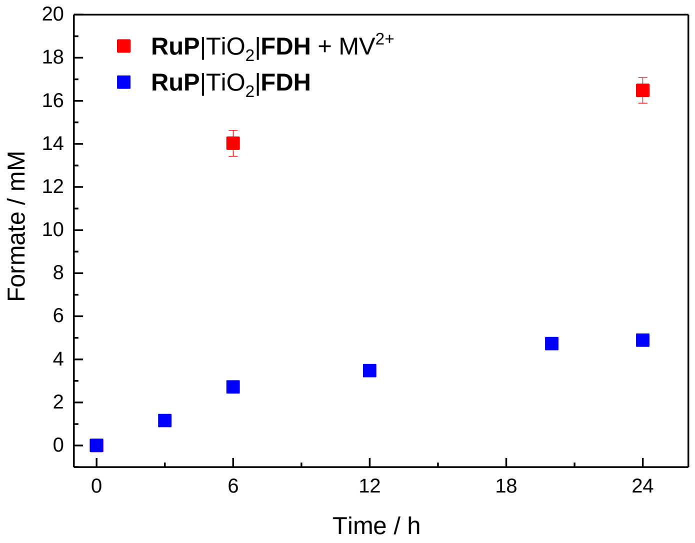

Figure S7. The effect of the addition of MV^{2+}, which can act as a mediator between TiO_2 and **FDH**, in a colloidal dye-sensitized TiO_2 system for photocatalytic CO_2 reduction to formate with **FDH**. Conditions: 12 nM **FDH**, 10 mM DTT, 2 mM MV^{2+}, 0.83 mg mL^{-1} TiO_2, 16.7 µM **RuP**, 100 mM TEOA, 100 mM $NaHCO_3$, pH 6.5, 25°C, N_2 atmosphere, total volume: 1.0 mL, simulated solar light irradiation: 100 mW cm^{-2}, AM 1.5G, $\lambda > 420$ nm. Error bars correspond to sample standard deviation (N = 3). Data for **RuP**|TiO_2|**FDH** (blue) up to 24 h was reproduced from Figure 4 and error bars are not visible because of the larger scale of the y-axis.

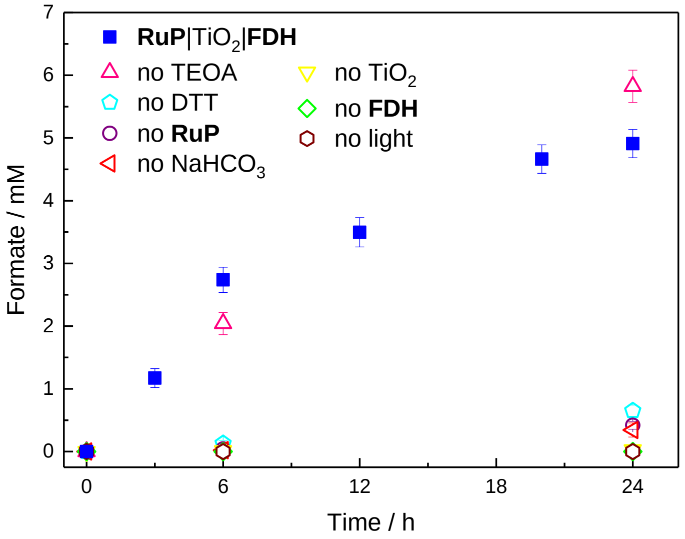

Figure S8. The change in formate concentration over time in colloidal dye-sensitized TiO_2 systems for photocatalytic CO_2 reduction to formate with **FDH**. Conditions: 12 nM **FDH**, 10 mM DTT, 0.83 mg mL^{-1} TiO_2, 16.7 µM **RuP**, 100 mM TEOA, 100 mM $NaHCO_3$, pH 6.5, 25°C, N_2 atmosphere, total volume: 1.0 mL, simulated solar light irradiation: 100 mW cm^{-2}, AM 1.5G, $\lambda > 420$ nm. Conditions omitted in different experiments as indicated in the figure key. Error bars correspond to sample standard deviation (N = 3). Data for **RuP**|TiO_2|**FDH** (blue) and for **RuP**|TiO_2 (no **FDH**, green) were reproduced from Figure 4. DTT can also act as an electron donor in the system (see Figure S9). The presence of a sacrificial donor like TEOA might also inhibit the reaction by forming degradation products,[9] which might be a reason for the higher activity in the absence of TEOA.

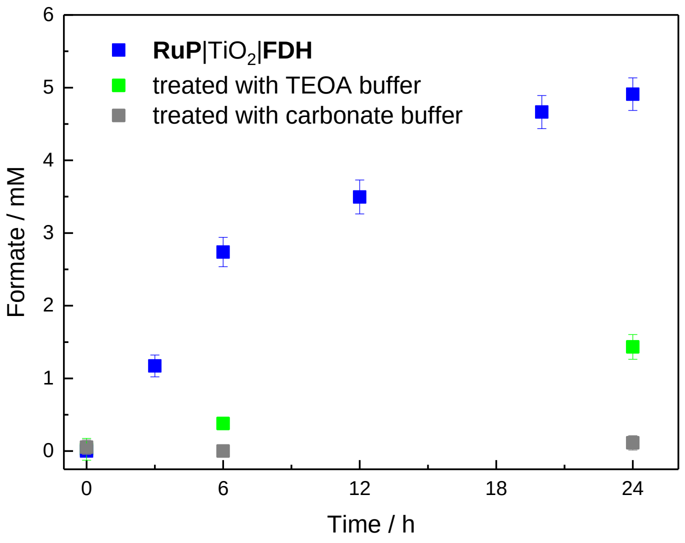

Figure S9. The effect of removing DTT from solution in a colloidal dye-sensitized TiO_2 system for photocatalytic CO_2 reduction to formate with **FDH**. TiO_2 particles with adsorbed **RuP** and **FDH** (**RuP**|TiO_2|**FDH**) were isolated after the assembly of a standard sample and the solution containing TEOA, $NaHCO_3$, and DTT was removed by three centrifugation and washing steps. Washing in carbonate buffer (100 mM, pH 6.5, grey) resulted in no formate production after 24 h, because no electron donor was present. In contrast, activity was observed when TEOA (100 mM, pH 6.5, green) was included in the washing solution. The activity was found to be lower, which is likely due to sample degradation from the mechanical stress during the washing procedure. Conditions: 12 nM **FDH**, 10 mM DTT, 0.83 mg mL^{-1} TiO_2, 16.7 µM **RuP**, 100 mM TEOA, 100 mM $NaHCO_3$, pH 6.5, 25°C, N_2 atmosphere, total volume: 1.0 mL, simulated solar light irradiation: 100 mW cm^{-2}, AM 1.5G, λ > 420 nm. Error bars correspond to sample standard deviation (N = 3). Data for **RuP**|TiO_2|**FDH** was reproduced from Figure 4.

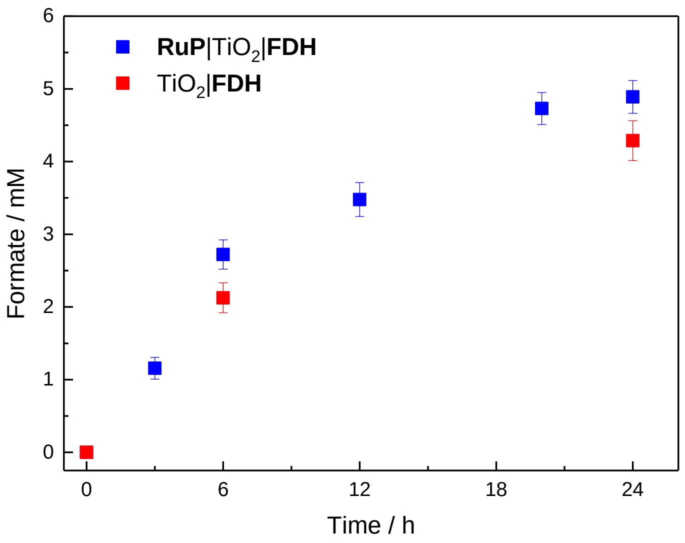

Figure S10. The change in formate concentration over time in a colloidal dye-sensitized TiO_2 system for photocatalytic CO_2 reduction to formate with **FDH** without **RuP** and full solar light irradiation (red). Conditions: 12 nM **FDH**, 10 mM DTT, 0.83 mg mL^{-1} TiO_2, 100 mM TEOA, 100 mM $NaHCO_3$, pH 6.5, 25°C, N_2 atmosphere, total volume: 1.0 mL, simulated solar light irradiation: 100 mW cm^{-2}, AM 1.5G (UV-vis spectrum irradiation). Error bars correspond to sample standard deviation (N = 3). Data for **RuP**|TiO_2|**FDH** (blue) was reproduced from Figure 4. Irradiation of **FDH**-modified TiO_2 without dye-sensitisation using the full solar spectrum (including band-gap excitation of TiO_2) results in comparable production of formate as with **RuP**|TiO_2|**FDH** over 24 h (Figure 4). Thus, conduction band electrons in TiO_2 can reduce CO_2 with **FDH**.

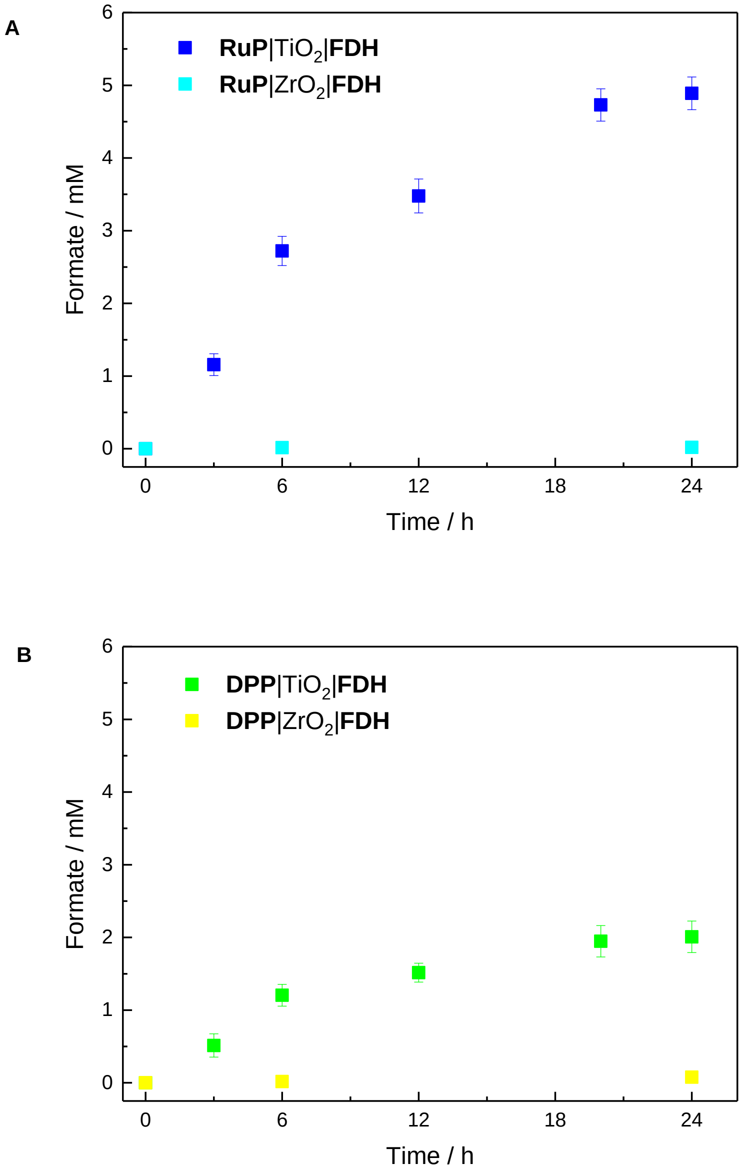

Figure S11. The effect of replacing TiO_2 with ZrO_2 in a colloidal dye-sensitized metal-oxide system for photocatalytic CO_2 reduction to formate with **FDH** with (A) **RuP** and (B) **DPP**. Conditions: 12 nM **FDH**, 10 mM DTT, 0.83 mg mL^{-1} TiO_2 / ZrO_2, 16.7 µM **RuP** / **DPP**, 100 mM TEOA, 100 mM $NaHCO_3$, pH 6.5, 25°C, N_2 atmosphere, total volume: 1.0 mL, simulated solar light irradiation: 100 mW cm^{-2}, AM 1.5G, λ > 420 nm. Error bars correspond to sample standard deviation (N = 3). Data for (A) **RuP** |TiO_2|**FDH** (blue) and (B) **DPP** |TiO_2|**FDH** (green) was reproduced from Figure 4. Replacing TiO_2 by ZrO_2, which cannot accept electrons from the dye due to its high conduction band energy (E_{CB}(ZrO_2)= −1.38 V *vs.* SHE at pH 6.5),[1] did not result in formate production. This suggests that electron transfer in dye|TiO_2|**FDH** occurs through the particle *via* the CB of TiO_2 rather than the surface of the semiconductor or directly from the dye to **FDH** as would be required for dye|ZrO_2|**FDH**.[1,10]

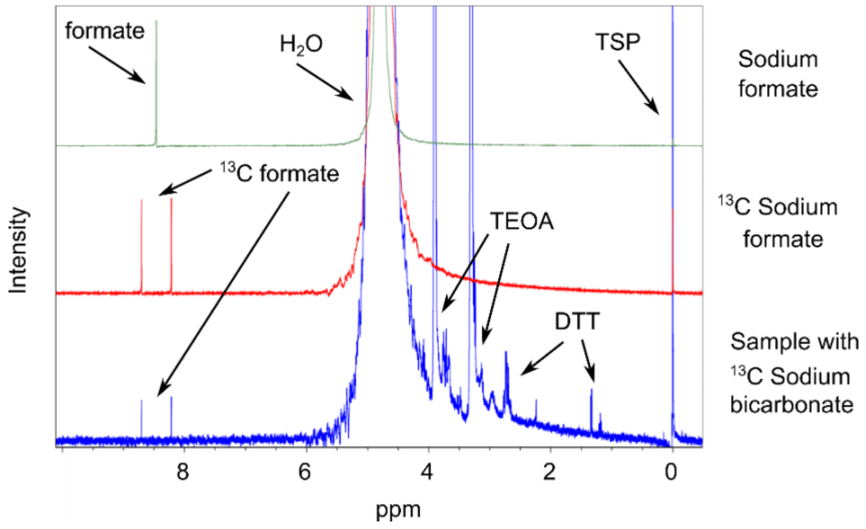

Figure S12. ^1H NMR spectrum (in D_2O containing 1.5 mM TSP) of HCO_2Na (green), $H^{13}CO_2Na$ (red), and sample (blue) of photocatalytic CO_2 reduction to formate with **FDH** in a colloidal dye-sensitized TiO_2. Conditions: 12 nM **FDH**, 10 mM DTT, 0.83 mg mL^{-1} TiO_2, 16.7 µM **RuP**, 100 mM TEOA, 100 mM $NaH^{13}CO_3$, pH 6.5, 25°C, N_2 atmosphere, total volume: 1.0 mL, simulated solar light irradiation: 100 mW cm^{-2}, AM 1.5G, λ > 420 nm. The photocatalysis experiment with $H^{13}CO_3Na$ confirmed that all observed formate was produced from CO_2.

Complete List of Authors from Main Text

Reference [8]: S. F. Rowe, G. Le Gall, E. V. Ainsworth, J. A. Davies, C. W. J. Lockwood, L. Shi, A. Elliston, I. N. Roberts, K. W. Waldron, D. J. Richardson, T. A. Clarke, L. J. C. Jeuken, E. Reisner, J. N. Butt.

Reference [19]: D. H. Nam, J. Z. Zhang, V. Andrei, N. Kornienko, N. Heidary, A. Wagner, K. Nakanishi, K. P. Sokol, B. Slater, I. Zebger, S. Hofmann, J. C. Fontecilla-Camps, C. B. Park, E. Reisner.

Supporting References

[1] J. Warnan, J. Willkomm, J. N. Ng, R. Godin, S. Prantl, J. R. Durrant, E. Reisner, *Chem. Sci.* **2017**, *8*, 3070–3079.

[2] E. Bae, W. Choi, *J. Phys. Chem. B* **2006**, *110*, 14792–14799.

[3] S. M. da Silva, C. Pimentel, F. M. A. Valente, C. Rodrigues-Pousada, I. A. C. Pereira, *J. Bacteriol.* **2011**, *193*, 2909–2916.

[4] K. P. Sokol, W. E. Robinson, A. R. Oliveira, J. Warnan, M. M. Nowaczyk, A. Ruff, I. A. C. Pereira, E. Reisner, *J. Am. Chem. Soc.* **2018**, *140*, 16418–16422.

[5] T. E. Rosser, M. A. Gross, Y.-H. Lai, E. Reisner, *Chem. Sci.* **2016**, *7*, 4024–4035.

[6] M. Kato, T. Cardona, A. W. Rutherford, E. Reisner, *J. Am. Chem. Soc.* **2012**, *134*, 8332–8335.

[7] P. G. Hoertz, Z. Chen, C. A. Kent, T. J. Meyer, *Inorg. Chem.* **2010**, *49*, 8179–8181.

[8] J. Krýsa, M. Baudys, M. Zlámal, H. Krýsová, M. Morozová, P. Klusoň, *Catal. Today* **2014**, *230*, 2–7.

[9] B. C. M. Martindale, E. Joliat, C. Bachmann, R. Alberto, E. Reisner, *Angew. Chemie Int. Ed.* **2016**, *55*, 9402–9406; *Angew. Chem.* **2016**, *128*, 9548–9552.

[10] M. A. Gross, A. Reynal, J. R. Durrant, E. Reisner, *J. Am. Chem. Soc.* **2014**, *136*, 356–366.

End of Supporting Information
